# Genomic and *in-vitro* characteristics of a novel strain *Lacticaseibacillus chiayiensis* AACE3 isolated from fermented blueberry

**DOI:** 10.3389/fmicb.2023.1168378

**Published:** 2023-05-19

**Authors:** Xin-Dong Li, Yi-Cen Lin, Rui-Si Yang, Xin Kang, Wei-Gang Xin, Feng Wang, Qi-Lin Zhang, Wen-Ping Zhang, Lian-Bing Lin

**Affiliations:** ^1^Faculty of Life Science and Technology, Kunming University of Science and Technology, Kunming, Yunnan, China; ^2^Engineering Research Center for Replacement Technology of Feed Antibiotics of Yunnan College, Kunming, Yunnan, China

**Keywords:** *Lacticaseibacillus chiayiensis*, whole-genome sequencing, comparative genomics, antimicrobial activity, antioxidation

## Abstract

Numerous different species of LAB are used in different fields due to their unique characteristics. However, *Lacticaseibacillus chiayiensis*, a newly established species in 2018, has limited microorganism resources, and lacks comprehensive evaluations of its properties. In this study, *L. chiayiensis* AACE3, isolated from fermented blueberry, was evaluated by genomic analysis and *in vitro* assays of the properties. The genome identified genes associated with biofilm formation (*luxS*, *ccpA*, *brpA*), resistance to oxidative stress (*tpx*, *trxA*, *trxB*, *hslO*), tolerance to acidic conditions (*dltA*, *dltC*), resistance to unfavorable osmotic pressure (*opuBB*, *gbuA*, *gbuB*, *gbuC*), and adhesion (*luxS*, *dltA*, *dltC*). The AACE3 showed 112 unique genes, relative to the other three *L. chiayiensis* strains. Among them, the presence of genes such as *clpP*, *pepO*, and *feoA* suggests a possible advantage of AACE3 over other *L. chiayiensis* in terms of environmental adaptation. *In vitro* evaluation of the properties revealed that AACE3 had robust antibacterial activity against eight common pathogens: *Streptococcus agalactiae*, *Staphylococcus aureus*, *Escherichia coli*, *Salmonella enteritidis*, *Salmonella choleraesuis*, *Shigella flexneri*, *Pseudomonas aeruginosa*, and *Klebsiella pneumoniae*. In addition, AACE3 showed more than 80% survival rate in all tests simulating gastrointestinal fluid, and it exhibited high antioxidant capacity. Interestingly, the cell culture supernatant was superior to intact organisms and ultrasonically crushed bacterial extracts in all tests of antioxidant capacity. These results suggested that the antioxidant capacity may originate from certain metabolites and extracellular enzymes produced by AACE3. Moreover, AACE3 was a moderate biofilm producer due to the self-agglomeration effect. Taken together, *L. chiayiensis* AACE3 appears to be a candidate strain for combating the growing incidence of pathogen infections and antioxidant production.

## 1. Introduction

Lactic acid bacteria (LAB) can produce a variety of extracellular polysaccharides, antimicrobial compounds and short-chain fatty acids, and have the advantage of being non-pathogenic, acid tolerant and bile tolerant (Mojgani et al., 2015; Vasconcelos et al., 2019). Several studies have confirmed that LAB have a variety of applications such as use in food fermentation to enhance food flavor, as food additives to promote host health, while LAB-produced bacteriocins are used in food preservation and antimicrobial therapy (Gerritsen et al., 2011; Kok and Hutkins, 2018; Xiang et al., 2022; Sharma et al., 2023). For example, the levels of organic acids, ethyl acetate and aromatic alcohols were increased and the organoleptic quality of the juice was improved by fermenting goji berry juice with *Lactobacillus paracasei* (Duan et al., 2023). During the livestock industry, the supplementation of diets with *Lactobacillus acidophilus* significantly reduced the abundance of potentially pathogenic *Escherichia coli* and increased the number of beneficial microorganisms in broilers intestine. The level of ileal amino acid digestion and humoral immunity were also increased (Zhang and Kim, 2014). In addition, *Lactobacillus salivarius* produced bacteriocin *XJS01* showed excellent therapeutic and healing-promoting effects against *Staphylococcus aureus* infection in mouse skin wounds (Xiang et al., 2022). Thus, the application and exploitation of functional LAB strains in the food fermentation industry, livestock industry and other related industries has greatly increased over the years (De Melo Pereira et al., 2018). However, more studies have emerged that focus on the rational improvement of industrially useful LAB strains (Zhang et al., 2022). Therefore, more precise confirmation of the phenotypes of novel LAB strains will certainly be required before their adoption for better application purposes.

LAB must meet certain well-known criteria to fulfill their intended functions and specific applications. For example, they must be able to withstand harsh conditions such as acidity, bile salts, and proteases in the human and animal digestive tracts (Ticho et al., 2019). They should also be capable of colonizing and adhering to diverse environments, which typically depends on self-aggregation and hydrophobic properties, and is reflected in biofilm formation (Lo Curto et al., 2011). Additionally, they must be able to produce antibacterial substances like bacteriocins to combat pathogens, and should have precise taxonomy and be easily identifiable for accurate application (Vizoso Pinto et al., 2006; Sanders et al., 2010). Notably, functional genomics analysis of LAB can ensure the accurate classification of strains and provide information on genetic features, metabolic capacity, and the correlation between genotypes and phenotypes (Guinane et al., 2016; Li et al., 2017). In the post-genomic era, the selection of LAB has relied heavily on phenotypic characterization to meet the needs of applications in different fields. Therefore, technological advances in functional genomics can help to rapidly identify new isolates and evaluate their performance.

*Lacticaseibacillus chiayiensis*, a new lactobacillus species, was first isolated from cattle manure collected in Chiayi city (Taiwan, China) in 2018 (Huang et al., 2018). *L. chiayiensis*, which is closely related to *L. casei* and *Lactobacillus zeae* in terms of genomics (Kim et al., 2021). To date, only three *L. chiayiensis* strains have been reported with genomic information, only one *L. chiayiensis* strain has a complete reported genome, and few studies have integrated functional genomic data with the properties of *L. chiayiensis* (Kim et al., 2021). Therefore, it is critically important to build a comprehensive profile of the characteristics and genomic information of newly isolated *L. chiayiensis* strains.

In this study, whole-genome sequencing was conducted on *L. chiayiensis* AACE3 to evaluate the organism’s potential properties at the genomic level. In addition, the genomic variations between the AACE3 strain and other *L. chiayiensis* strains were examined by comparative genomic analysis. Finally, the *in-vitro* characteristics of *L. chiayiensis* AACE3 were experimentally evaluated, including its stress tolerance in a simulated gastrointestinal environment, antagonism profile against pathogenic bacteria, biofilm formation behavior and antioxidant capacity. These findings provide new perspectives for the comprehensive evaluation of newly isolated LAB.

## 2. Materials and methods

### 2.1. Bacterial strains and culture conditions

*Lacticaseibacillus chiayiensis* AACE3 was isolated from fermented blueberry. LAB were routinely grown in the study in MRS broth at 37°C for 24 h and reinoculated in fresh MRS broth at 37°C. The pathogens used in this study and their growth conditions are presented in Table 1. All the strains used in this study are stored at the Faculty of Life Science and Technology, Kunming University of Science and Technology, Kunming city, China.

**Table 1:** Antibacterial spectrum of *Lacticaseibacillus chiayiensis* AACE3.

Strains of pathogenic bacteria	Source^a^	Medium and temperature (°C)	Diameter of bacteriostatic circle(mm)^b^
Gram-positive bacteria			
*Streptococcus agalactiae*	CMCC(B)32,116	BHI,37	24.35 ± 0.44
*Staphylococcus aureus*	ATCC6538	LB,37	21.05 ± 0.13
Gram-negative bacteria			
*Escherichia coli*	CMCC(B)44,102	LB,37	17.96 ± 0.22
*Salmonella enteritidis*	CMCC(B)50,335	LB,37	18.22 ± 0.14
*Salmonella choleraesuis*	ATCC13312	LB,37	14.67 ± 0.84
*Shigella flexneri*	CMCC(B)51,572	LB,37	15.17 ± 0.34
*Pseudomonas aeruginosa*	ATCC27853	LB,37	16.98 ± 0.35
*Klebsiella pneumoniae*	ATCC10031	BHI,37	19.46 ± 0.28

^a^ATCC, American Type CultureCollection; CMCC, China Center of Medicine Culture Collection. ^b^Values are the means of three experiments in duplicate; ±, indicates standard deviation.

### 2.2. Whole-genome sequencing, assembly, and annotation

Total genomic DNA was extracted by SDS extraction combined with column purification. DNA purity and quantity were confirmed spectrophotometrically at 260 nm using a NanoDrop One spectrophotometer (NanoDrop Technologies, Wilmington, DE). A combination of the Illumina platform (Illumina, TX, USA) and Oxford Nanopore platform (Oxford Nanopore Technologies, Oxford, UK) was used to sequence the genomes. After filtering low-quality and short-length reads from raw sequencing data, the reads were assembled using Unicycler V0.4.9 (Wick et al., 2017). Prokka V1.12 (Seemann, 2014) was utilized to predict protein-coding RNA for the assembled genome. The prediction of rRNA and tRNA sequences was performed by RNAmmer V1.2 (Lagesen et al., 2007) and Aragorn V1.2 (Laslett and Canback, 2004), respectively. Functional gene annotation was performed based on clusters of orthologous groups (COGs) (Galperin et al., 2015). The identification of secondary metabolite biosynthesis gene clusters was conducted with antiSMASH version 5.1.2 (Blin et al., 2019). The bacteriocin synthesis gene cluster of the strain was analyzed with BAGEL4 (van Heel et al., 2018).

### 2.3. Comparative genomic analysis

For comparative genomic and phylogenetic analyses, genomic data of *L. chiayiensis* and genetic information of other LAB strains were retrieved from the NCBI database,^1^ and the genomic information is shown in Supplementary Table S1. Average nucleotide identity (ANI) analysis was performed for the complete genome assembly using FastANI v2.0 (Jain et al., 2018). The 16S rRNA gene sequence was extracted from the whole-genome sequence, and a phylogenetic tree was constructed in MEGA-X using the neighbor-joining method. Initially, the ClustalW multiple sequence comparison method is selected for alignment, and then the Neighbor-Joining Tree is constructed. The Substitution model for calculating the genetic distance used the Maximum Composite Likelihood. The columns with more null positions in the multiple sequence comparison are processed by pairwise deletion. BPGA (Bacterial Pan Genome Analysis tool v. 1.3) was used to analyze the pangenome and core genome of the strains with default parameters (Chaudhari et al., 2016), and core gene evolutionary trees were constructed to infer the evolutionary relationships of the strains. The phylogenetic tree based on the core genes was constructed in BPGA (Bacterial Pan Genome Analysis tool v. 1.3) software using the neighbor joining method with default parameters. The “core genome” is typically defined as a set of genes shared by all strains (Lawrence and Hendrickson, 2005). COG functional classification assignment of core genomic and pangenomic genes of the AACE3 strain using USEARCH in the BPGA tool. Unique genes of AACE3 were annotated by using the KEGG database for pathway analysis.

### 2.4. Assessment of *in vitro* characteristics

#### 2.4.1. Antibacterial activity

The antibacterial activity and antimicrobial spectrum of *L. chiayiensis* AACE3 against gram-positive (2) and gram-negative (6) indicators were determined by the modified Oxford cup double-layer plate method (Voulgari et al., 2010). Briefly, *L. chiayiensis* AACE3 was inoculated in MRS broth and cultured at 37°C for 24 h. The obtained fresh cultures were centrifuged at 8000 × *g* at 4°C for 15 min in a high-speed refrigerated centrifuge (Thermo Scientific, Waltham, MA, USA). The cell-free supernatant (CFS) was sterilized using a 0.22 μm filter membrane before use. Then, the indicated pathogens (100 μL, 1 × 10^7^ CFU/mL) were mixed with 5 mL of semisolid LB agar medium and quickly poured onto the surface of solid LB agar medium. The Oxford cups were placed on the agar surface, 200 μL of CFS was added, and the plates were allowed to stand at 4°C until the CFS had fully diffused. Then, the plates were transferred to a constant-temperature incubator at 37°C for 24 h to observe the bacterial inhibitory activity.

#### 2.4.2. Tolerance to simulated gastrointestinal conditions and bile salts

*In vitro* simulated gastrointestinal fluid and bile salt tolerance models were developed based on previous studies with modifications (Zhao et al., 2021). The artificial gastric juice was prepared by mixing 10 g/L pepsin (Solarbio, Beijing, China) with 16.4 mL of 0.1 mol/L HCl, and the pH was adjusted to 2.0, 3.0, and 4.0 with 1 mol/L NaOH. The prepared solutions were sterilized using a 0.22 μm filter membrane before use. For the artificial intestinal juice preparation, 10.0 g/L trypsin (Solarbio, Beijing, China) and 6.8 g KH_2_PO_4_ were mixed in 500 mL of sterile ddH_2_O, and the pH was adjusted to 6.8 with 1 mol/L NaOH; the solution was then filtered through a 0.22 μm membrane. The LAB suspension was inoculated in 5 mL of simulated intestinal juice and simulated gastric juice at pH 2.0, 3.0 and 4.0 and incubated at 37°C for 3 and 4 h. The LAB suspension was inoculated in MRS broth containing 0.3% bile salts and cultured at 37°C for 24 h. The survival rate was calculated using the plate count method with the following formula:


$$\mathrm{Survival rate}\;\left(\%\right)=\mathrm{lg}\;N_{1}/\mathrm{lg}\;N_{0}\times 100\%$$

where N_0_ is the total number of viable bacteria in the untreated group, and N_1_ is the total number of viable bacteria after treatment with artificial gastrointestinal juice or bile salts.

#### 2.4.3. Autoaggregation

The autoaggregation tests were performed according to a previously described method (Kim et al., 2021). Briefly, bacterial cultures were centrifuged and resuspended in PBS, and the absorbance at 600 nm (OD_600_, A_0_) was adjusted to 0.6. The adjusted mixture was shaken and vortexed for 20 s and then incubated at room temperature for 3 h and 24 h. The absorbance of the supernatant at 600 nm was measured at 3 h and 24 h (A_t_). The autoaggregation was calculated with the following formula:


$$A\;\left(\%\right)=\left(A_{0}-A_{t}\right)/A_{0}\times 100\%$$

#### 2.4.4. Cell surface hydrophobicity

The cell surface hydrophobicity was determined as previously described (Fadare et al., 2022). Freshly cultured LAB strains were centrifuged and resuspended in PBS, and the absorbance at 600 nm (OD_0_) was adjusted to 0.8 ± 0.2. Then, 1 mL of ethyl acetate or xylene was added to 5 mL of LAB suspension and vortexed for 2 min. After incubation at 37°C for 1 h, the aqueous phase was collected, and the absorbance at 600 nm was measured. The hydrophobicity value was calculated according to the following formula:


$$H\;\left(\%\right)=\left({\mathrm{OD}}_{0}-\mathrm{OD}\right)/{\mathrm{OD}}_{0}\times 100\%$$

where OD_0_ and OD indicate the absorbance before and after mixing with ethyl acetate or xylene, respectively.

#### 2.4.5. Determination of biofilm formation

The biofilm formation of *L. chiayiensis* AACE3 was quantitatively determined following previously reported methods with minor modifications (Benmouna et al., 2020). The bacterial cultures (grown for 24 h) were washed twice with PBS and resuspended to an OD_600_ of 1.0. Fifty microliters of bacterial suspension and 150 μL sterile MRS broth were added to a 96-well plate and incubated at 37°C for 24 h, while 200 μL of sterile MRS broth was used as a negative control. After 24 h, the medium in the wells was poured out, and sterile PBS was slowly added to the wells to wash the unattached bacteria; three repeat washes were performed. After drying at room temperature for 20 min, the biofilms were fixed with 10% methanol solution for 30 s. Then, 1% crystalline violet dye was used to stain the biofilms for 20 min, and the stained biofilms were washed three times with sterile water to remove excess stain. Finally, 200 μL of anhydrous ethanol was added to each well, and the optical density of the stained adnexal cells was measured at 595 nm. OD_t_ and OD_c_ represent the results of the experimental and control groups, respectively. The biofilm production capacity was measured as follows: OD_t_ ≤ OD_c_ indicated nonbiofilm producer (0), OD_c_ < OD_t_ ≤ 2ODc indicated a weak biofilm producer (+), 2OD_c_ < OD_t_ ≤ 4OD_c_ indicated a moderate biofilm producer (++), and OD_t_ > 4OD_c_ indicated a robust biofilm producer (+++).

### 2.5. Antioxidant activity

#### 2.5.1. Preparation of samples

The CFS of the strains, the washed and resuspended intact organism (IO), and the ultrasonically fragmented extract of the organism were used for the antioxidant viability test. The CFS was collected by centrifugation (8,000 × *g*, 15 min) of AACE3 (grown for 24 h) at 4°C. IOs were washed three times with PBS (pH 7.4) and resuspended in PBS at a concentration of 1.0 × 10^9^ CFU/mL. Ultrasonically crushed bacterial extracts (UCBEs) were obtained by ultrasonic crushing in an ice bath for 15 min, and the supernatant was obtained by centrifugation (8,000 × *g*, 15 min) at 4°C. The CFS and UCBE were filtered with 0.22 μm filter membranes and used for further experiments.

#### 2.5.2. Hydroxyl radical scavenging

The hydroxyl radical scavenging assay was performed as previously reported (Oyaizu, 1988). Then, 2.5 mmol/L 1,10-phenanthroline (1.0 mL), 2.5 mmol/L FeSO_4_ (1.0 mL), and PBS (1.0 mL, pH 7.4) were added to 1 mL of each sample. After thorough mixing, 20 mmol/L H_2_O_2_ (1 mL) was added, and the sample was placed in a constant-temperature water bath at 37°C for 90 min, and the absorbance at 517 nm was measured. The hydroxyl radical scavenging rate was calculated by the following formula:


$$\mathrm{Scavenging rate}\;\left(\%\right)=\left[\left(A_{s}-A_{n}\right)/\left(A_{b}-A_{n}\right)\right]\times 100\%$$

The absorbance value of the samples measured in the experimental group was A_s_. Distilled water was used instead of the experimental sample in a blank group to measure the absorbance value A_b_, and distilled water was used instead of H_2_O_2_ to measure the absorbance value A_n_.

#### 2.5.3. DPPH radical scavenging

The measurement of DPPH radical scavenging capacity was based on a previous study with minor adjustments (Lin and Yen, 1999). One milliliter of preprepared sample was mixed with 1 mL of 0.2 mmol/L DPPH solution and placed in the dark for 30 min, and the absorbance at 517 nm was measured. The DPPH radical scavenging rate was calculated by the following formula:


$$\mathrm{Scavenging rate}\;\left(\%\right)=\left[1-\left(A_{s}-A_{b}\right)/A_{c}\right]\times 100\%$$

The absorbance value of the samples measured in the experimental group was A_s_. Distilled water was used instead of the experimental sample as a control group to measure the absorbance value A_c_, and absolute alcohol was used instead of H_2_O_2_ to measure the absorbance value A_b_.

#### 2.5.4. Superoxide anion scavenging ability

The superoxide anion radical scavenging performance was evaluated according to a previous study (Li et al., 2012). One milliliter of preprepared sample and 3.0 mL of Tris–HCl solution (pH 8.2, 50 mM) were mixed and placed at room temperature for 20 min. Then, 0.4 mL of 25 mmol/L pyrogallol was added, and the sample was placed in the dark for 4 min. Then, 0.5 mL of 8 mmol/L HCl was added to end the reaction. The absorbance at 325 nm was recorded. The superoxide anion radical scavenging rate was calculated by the following formula:


$$\mathrm{Scavenging rate}\;\left(\%\right)=\left(1-A_{s}/A_{b}\right)\times 100\%$$

where A_s_ represents the absorbance of each sample. The blank contained distilled water instead of the sample used in the reaction system.

### 2.6. Statistical analysis

All experimental data were obtained from at least three independent replicates and are expressed as the mean ± standard deviation (SD). The statistical significance between two groups was assessed by using the unpaired two-tailed t test by using GraphPad Prism 8.0.2 (GraphPad Software Inc., La Jolla, CA, USA). The statistically significant differences between multiple groups were analyzed by one-way ANOVA by using GraphPad Prism 8.0.2 (GraphPad Software Inc., La Jolla, CA, USA). The criterion for statistical significance was a *p* value <0.05.

## 3. Results

### 3.1. General genomic characteristics

**Figure 1:**
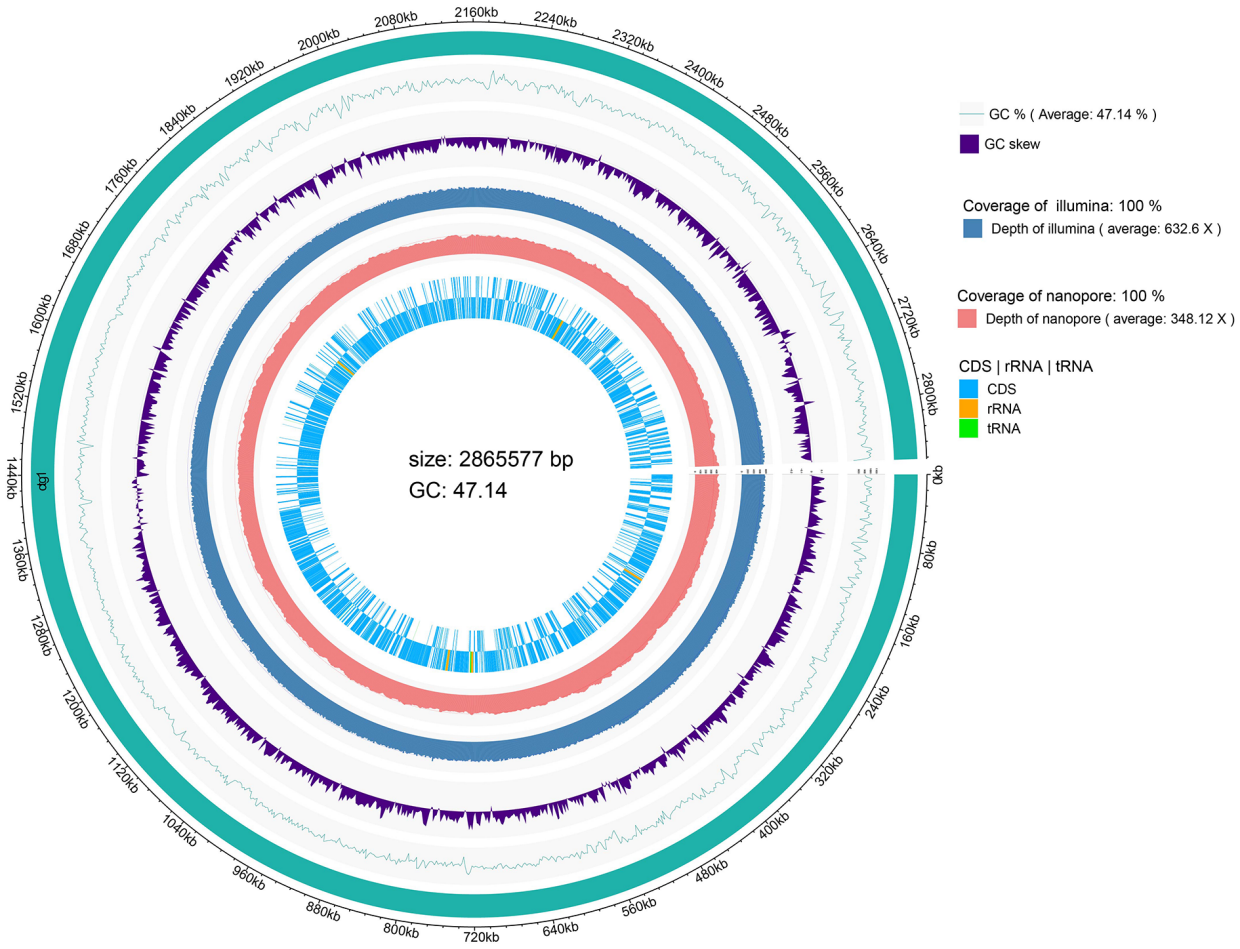
Circular genome map of *Lacticaseibacillus chiayiensis* AACE3.

The genome size of *L. chiayiensis* AACE3 was 2,865,577 bp, with an average GC content of 47.14% (Figure 1). A total of 2,758 protein-coding sequences (CDSs) were identified among 2,870 predicted genes. In addition, 261 pseudogenes, 58 tRNAs, 15 rRNA manipulators (including 5 23S rRNAs, 5 16S rRNAs, 5 5S rRNAs) and 3 clustered regularly interspaced short palindromic repeat (CRISPR) sequences were predicted in the genome. All protein domains were classified into 21 functional categories according to the COG database, mainly carbohydrate transport and metabolism (G, 206), translation/ribosome structure and biogenesis (J, 145), amino acid transport and metabolism (E, 118), cell wall/membrane/envelope biogenesis (M, 80) and replication/recombination/repair (L, 79) (Supplementary Figure S1). The whole-genome sequence of *L. chiayiensis* has been submitted to GenBank with the accession number CP107523.1.

### 3.2. Phylogenetic analysis of *Lacticaseibacillus chiayiensis*

**Figure 2:**
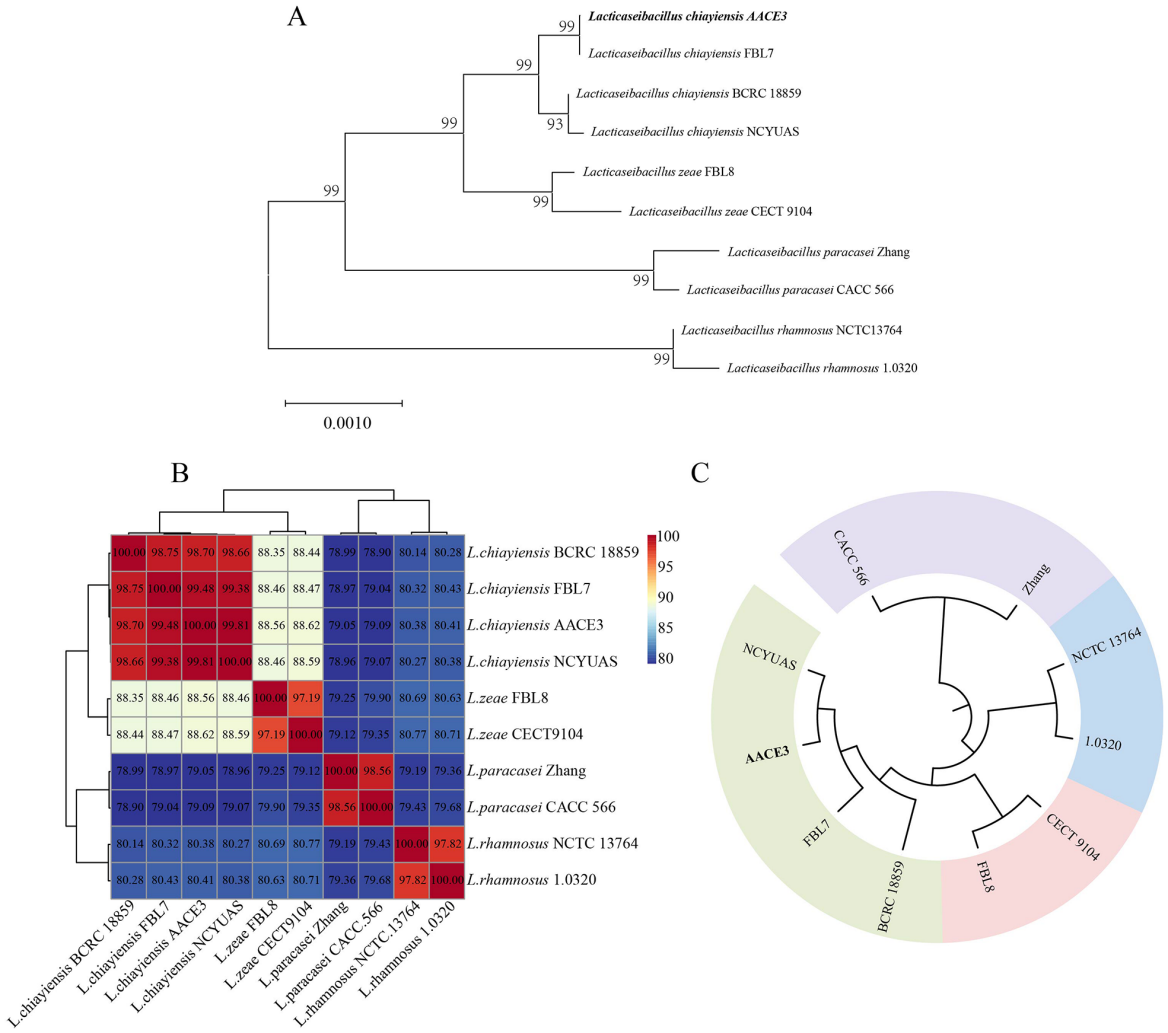
Phylogenetic analysis of *L. chiayiensis* AACE3 based on **(A)** 16S rRNA gene sequences and **(B)** average nucleotide identity **(C)** of the core genome.

The phylogenetic relationships of *L. chiayiensis* AACE3 with other related species were analyzed. The phylogenetic tree was constructed based on 16S rRNA sequences, which showed that the *L. chiayiensis* AACE3 strain shared 99.86–100% identity with other *L. chiayiensis* isolates (Figure 2A). However, the identity with *L. zeae*, *Lacticaseibacillus paracasei*, and *Lacticaseibacillus rhamnosus* was more than 98%, exceeding the species classification threshold of 97% for 16S rRNA (Supplementary Table S2), indicating failure to effectively detect species-specific differences. The ANI assay between the genomes of *L. chiayiensis* AACE3 and other strains clearly distinguished *L. chiayiensis* from *L. zeae*, *L. paracasei*, and *L. rhamnosus* (Figure 2B). The genome of *L. chiayiensis* AACE3 was most similar to that of *L. chiayiensis* NCYUAS (99.81%) compared to those of other *L. chiayiensis* strains. In addition, in a core genome-based phylogenetic analysis, the evolutionary relationship between strain AACE3 and strain NCYUAS was shown to be the closest, with this result being similar to that obtained by ANI analysis (Figure 2C).

### 3.3. Genomic synteny and pangenomic and core genome analyses

**Figure 3:**
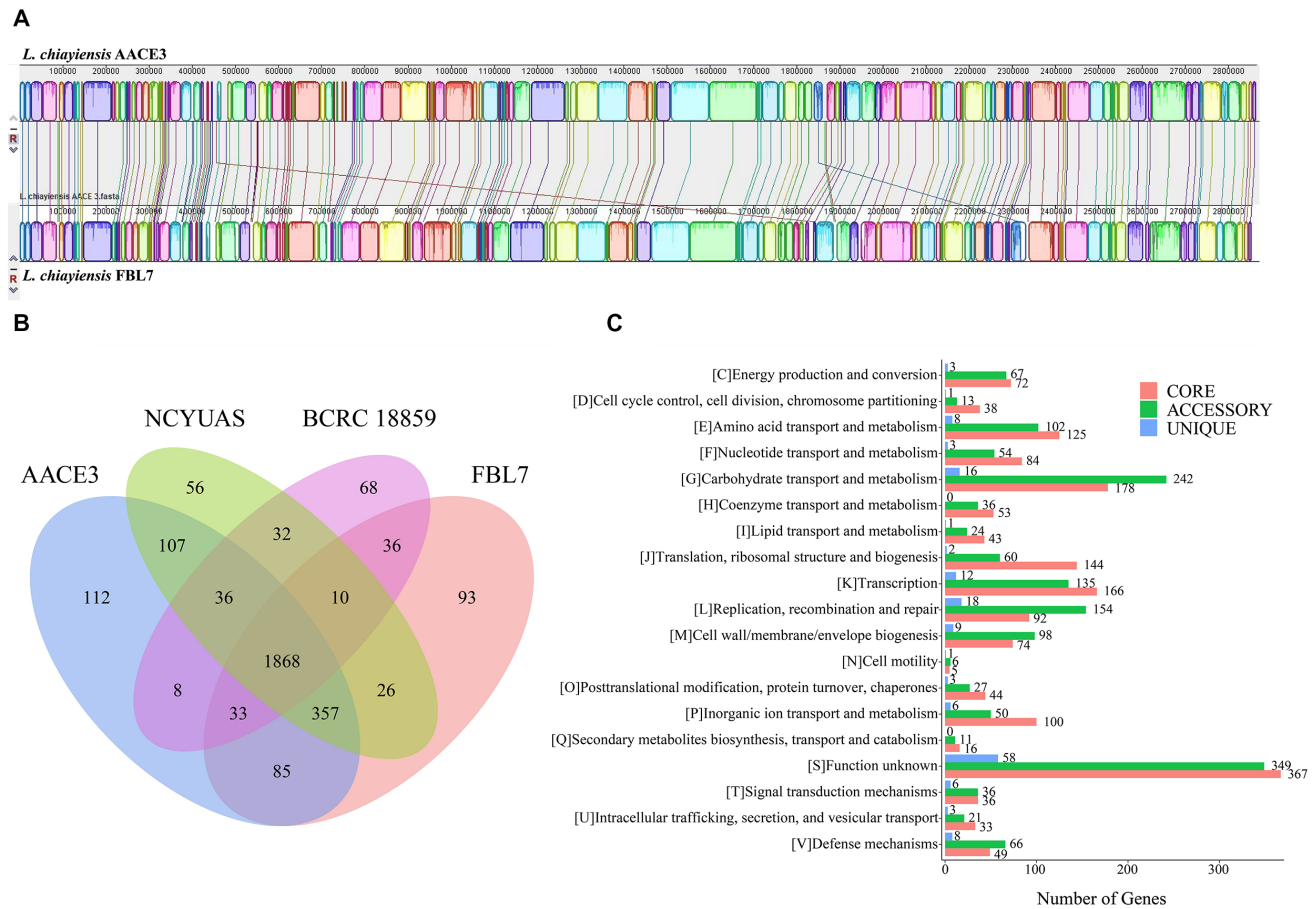
Comparative genomic analysis of *L. chiayiensis* AACE3 with three closely related *Lacticaseibacillus* species. **(A)** Genome-to-genome alignment of *L. chiayiensis* AACE3 and *L. chiayiensis* FBL7 using a progressive mauve software with a window of 1,000 nucleotides. Boxes with the same color indicate the syntenic regions. **(B)** Venn diagram showing the number of genes of orthologous CDSs shared and unique among the three strains *L. chiayiensis* AACE3, *L. chiayiensis* FBL7, *L. chiayiensis* NCYUAS and *L. chiayiensis* BCRC 18859. **(C)** The number of core, accessory, and unique genes among *L. chiayiensis* genomes assigned in cluster of orthologous group (COG) categories.

To explore the genetic differences between the *L. chiayiensis* strains, *L. chiayiensis* FBL7 and *L. chiayiensis* AACE3, which were selected as complete at the genomic level, were analyzed for genomic synteny. As shown in Figures 3A, a large homologous region between *L. chiayiensis* FBL7 and *L. chiayiensis* AACE3 was found, and the close relationship between the two strains was consistent.

Four pangenomes of the *L. chiayiensis* strains consisted of 1868 core genes, 1896 accessory genes and 329 unique genes (Figure 3B). Among them, 1719 genes in the core genome were assigned to 19 COG categories. Most core genes were assigned to the functional categories of carbohydrate transport and metabolism (G, 178), transcription (K, 166) and translation, ribosomal structure and biogenesis (J, 144) (Figure 3C).

To further investigate the genetic characteristics and functional properties of *L. chiayiensis* AACE3, unique genes of *L. chiayiensis* AACE3 were classified using the COG and KEGG annotation databases. The 112 unique genes belonging to *L. chiayiensis* AACE3 encoded 106 hypothetical proteins and 6 known proteins, of which only 28 unique genes were annotated by the COG database and were classified into 10 COG categories (Supplementary Table S3). Two genes were assigned to the defense mechanisms category, containing genes encoding ABC-type bacteriocin lantibiotic exporters (locus_01810). Two genes were assigned to the posttranslational modification, protein turnover, and chaperones category, and one gene was assigned to the intracellular trafficking, secretion, and vesicular transport category, containing genes encoding the ATP-dependent *clp* protease proteolytic subunit (locus_00468). Two genes were assigned to the inorganic ion transport and metabolism category, including the *feoA* (locus_01741) gene. In addition, unique genes, including the *lexA* repressor (locus_00434), a key regulator in the bacterial SOS response, were detected.

### 3.4. Annotation of genes coded by *Lacticaseibacillus chiayiensis* AACE3 that are implicated in stress response

Several proteins encoded in the *L. chiayiensis* AACE3 genome that are associated with tolerance to stress factors, such as unfavorable osmotic pressure (i.e., *anuBB*, *ghux*, *gbuB*, *gbuC*), and acidic environments (i.e., *dltA*, *dltC*), have been identified. In addition, the presence of *luxS*, *dltA*, and *dltC* genes in the genome was shown to be associated with bacterial adhesion and colonization. As well as genes responsible for resistance to oxidative stress *tpx*, *trxA*, *trxB* and *hslO* and biofilm formation *luxS*, *ccpA* and *brpA* genes were also identified (Table 2).

**Table 2:** Probiotic-related genes present in *L. chiayiensis* AACE3.

	Position	Product	Gene	Reference
Acid tolerance	locus_00678	D-alanine--poly(phosphoribitol) ligase subunit	*dltA*	Walter et al. (2007)
	locus_00680	D-alanine--poly(phosphoribitol) ligase subunit	*dltC*	Walter et al. (2007)
Oxidative stress	locus_00631	Thiol peroxidase	*tpx*	Chae et al. (1994)
	locus_00253	Thioredoxin	*trxA*	Dinarieva et al. (2023)
	locus_00893	Thioredoxin reductase	*trxB*	Dinarieva et al. (2023)
	locus_02410	33 kDa chaperonin protein HslO	*hslO*	Kaidow et al. (2022)
Osmotic pressure	locus_02498	Choline transport system permease protein	*opuBB*	Dupre et al. (2019)
	locus_02045	Glycine betaine/carnitine transport ATP-binding	*gbuA*	Sleator et al. (2003)
	locus_02044	Glycine betaine/carnitine transport permease	*gbuB*	Sleator et al. (2003)
	locus_02043	Glycine betaine/carnitine transport binding	*gbuC*	Sleator et al. (2003)
Adhesion	locus_00654	S-ribosylhomocysteine lyase	*luxS*	Ramić et al. (2023)
	locus_00678	D-alanine--poly(phosphoribitol) ligase subunit	*dltA*	Walter et al. (2007)
	locus_00680	D-alanine--poly(phosphoribitol) ligase subunit	*dltC*	Walter et al. (2007)
Biofilm formation	locus_00654	S-ribosylhomocysteine lyase	*luxS*	Ramić et al. (2023)
	locus_00693	Catabolite control protein A	*ccpA*	Zheng et al. (2022)
	locus_00237	Biofilm regulatory protein A	*brpA*	Wen and Burne (2002)

### 3.5. Secondary metabolite prediction analysis

**Figure 4:**
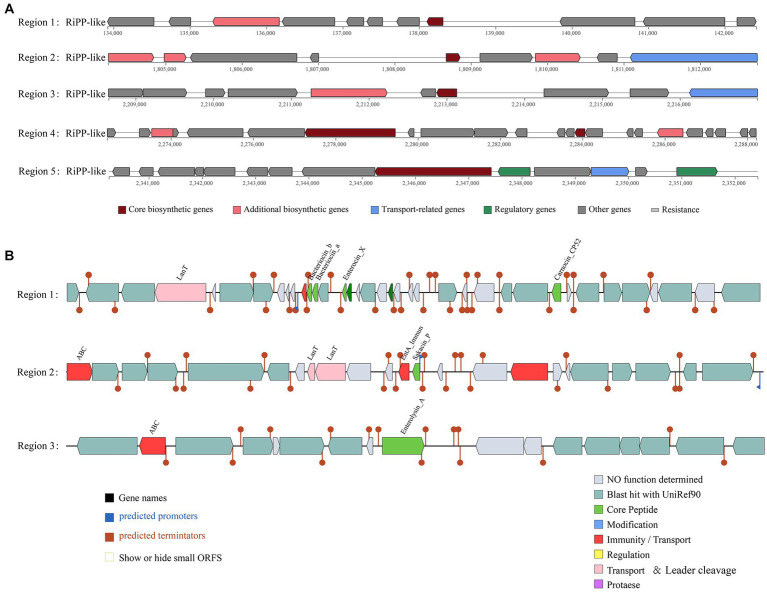
Prediction of antimicrobial activity-associated protein structures in the genome of *L. chiayiensis* AACE3. **(A)** Synthetic gene cluster of ribosomal synthesis and posttranslational modification peptide product (RiPP; based on antiSMASH database prediction). **(B)** Three bacteriocin (region_1: Enterocin_X, region_2: Sakacin_P and region_3: Enterolysin_A) synthesis gene clusters (predicted based on the BAGEL4 database).

According to the antiSMASH database, the strain has five ribosomal synthesis and posttranslational modification peptide product (RiPP) clusters, all of which may have the ability to produce bacteriocins (Figure 4A). Annotation of each gene for the five RiPP clusters is shown in Supplementary Table S4. The first and second RiPP-like clusters are based on the sakacin-P bacteriocin-encoding gene as the core biosynthetic gene. The core biosynthetic genes in the third and fourth clusters are *Blp* family class II bacteriocin genes. The fifth cluster is based on the ABC transporter protein as the core gene. In addition, unlike in other *L. chiayiensis* strains, ABC-type bacteriocin lantibiotic exporter-related protein genes and some hypothetical protein genes are present in the second and fourth clusters of *L. chiayiensis* AACE3.

The prediction of bacteriocin gene clusters from the BAGEL4 database showed that three bacteriocin synthesis gene clusters were present (Figure 4B). One cluster had enterocin_X, carnocin_CP52 and two antimicrobial peptides as core genes and included an immunity gene and a transport protein gene, and one had sakacin_P as a core gene and included three bacteriocin immunity/transport genes. The third cluster contained enterolysin_A as a core gene and an ABC transporter protein gene. In addition, several other genes related to bacteriocin secretion and processing and bacteriocin autoimmune genes such as *lagD* and *mccF* were also included in these three clusters (Supplementary Table S5).

### 3.6. Assessment of *in vitro* characteristics

#### 3.6.1. Antimicrobial activity of *Lacticaseibacillus chiayiensis* AACE3

Results for the inhibition activity of *L. chiayiensis* AACE3 against eight pathogenic strains are shown in Table 1. The results indicate that AACE3 has broad-spectrum antibacterial activity against common gram-positive and gram-negative pathogens. *L. chiayiensis* AACE3 showed the best inhibition activity against *Streptococcus agalactiae* CMCC (B) 32116 and *Staphylococcus aureus* ATCC 6538, with inhibition zone diameters of 24.35 ± 0.44 mm and 21.05 ± 0.13 mm, respectively. Moreover, AACE3 showed robust antibacterial activity against gram-negative indicators such as *Klebsiella pneumoniae* ATCC 10031 (inhibitory zone diameter of 19.46 ± 0.28 mm), *Salmonella enteritidis* CMCC (B) 50335 (inhibitory zone diameter of 18.22 ± 0.14 mm) and *Escherichia coli* CMCC (B) 44102 (inhibitory zone diameter of 17.96 ± 0.22 mm).

#### 3.6.2. Simulated gastrointestinal digestion and bile salt tolerance assay

Table 3 shows the survival rate of *L. chiayiensis* AACE3 under *in vitro* simulated gastrointestinal digestive juice conditions. AACE3 showed the highest tolerance to the intestinal juice, with a survival rate of 99.87%. The survival rates under gastric juice treatment under all three pH gradients were more than 80%, and the highest survival rate was 98.91% at pH 4.0. AACE3 had a survival rate of 84.32 ± 5.62% after 24 h of 0.3% bile salt treatment. In conclusion, *L. chiayiensis* AACE3 showed good tolerance to extreme environmental factors such as acidity, bile salts and proteases.

**Table 3:** Mean counts of *L. chiayiensis* AACE3 under exposure to simulated gastrointestinal fluid conditions.

Classification	Mean of viable count (log_10_ CFU/ mL) ± SD			Survival (%)
	Time of exposure (h)			
	0	3	4	
Artificial gastric juice (pH = 2.0)	7.32 ± 0.04	5.92 ± 0.13	——	80.87
Artificial gastric juice (pH = 3.0)		6.41 ± 0.23	——	87.57
Artificial gastric juice (pH = 4.0)		7.24 ± 0.11	——	98.91
Artificial intestinal juice	7.44 ± 0.12	——	7.43 ± 0.05	99.87

#### 3.6.3. Autoaggregation

The autoaggregation ability of strain AACE3 was examined, and the autoaggregation rate was 29.85 ± 2.56% at 3 h. After 24 h, the autoaggregation rate reached 61.25 ± 4.22%.

#### 3.6.4. Cell surface hydrophobicity and biofilm formation capacity

AACE3 showed a decrease in optical density in the presence of ethyl acetate, with a hydrophobicity index of 32.56 ± 4.85%. The biofilm formation ability of *L. chiayiensis* AACE3 was quantitatively assessed by crystalline violet staining. The results showed that the biofilm formation ability of the experimental group was 3.39-fold higher than that of the negative control, which was a moderate biofilm producer (++).

### 3.7. Antioxidant properties

**Figure 5:**
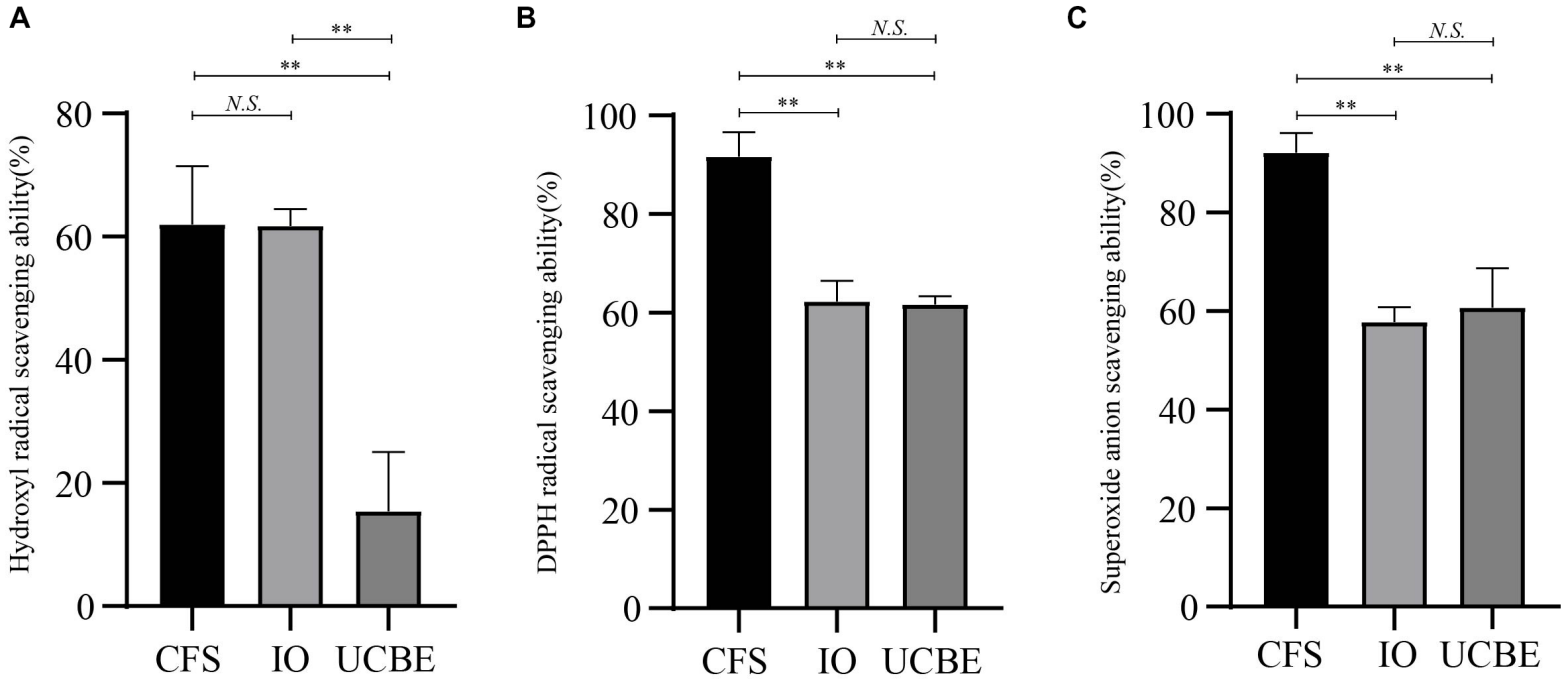
Antioxidant properties of *L. chiayiensis* AACE3. **(A)** Hydroxyl radical scavenging capacity, **(B)** DPPH radical scavenging capacity, **(C)** superoxide anion radical scavenging capacity. CFS, cell-free supernatant; IO, intact organisms; UCBE, ultrasonically crushed bacterial extract. **p < 0.01, *N.S.* denotes not significant.

The hydroxyl radical scavenging ability, DPPH radical scavenging ability and superoxide anion scavenging ability were analyzed to demonstrate the antioxidant capacity of strain AACE3 (Figure 5). The results showed that strain AACE3 exhibited good antioxidant capacity, and the CFS retained the highest antioxidant capacity. The DPPH radical scavenging ability and superoxide anion scavenging ability of the CFS were 91.67 ± 5.00% and 92.15 ± 3.97%, respectively. No significant difference between the resuspended intact organisms and crushed cell extracts was observed. In addition, the hydroxyl radical scavenging abilities of the supernatant (62.03 ± 9.39%) and resuspended IOs (61.79 ± 2.70%) were not significantly different.

## 4. Discussion

Discovery of new LAB and evaluation of their properties are crucial for meeting the needs for application in different fields. In this study, *L. chiayiensis* AACE3 isolated from fermented blueberry was comprehensively investigated by *in vitro* characterization and comparative genomic analysis. The genome size of strain AACE3 was 2,865,577 bp, and the GC content was 47.14%. In previous studies, it was reported that genome size and GC content can reflect the environmental adaptability of strains, with larger genome sizes indicating greater environmental adaptability (Bentkowski et al., 2015; Jia et al., 2022). Strains with large genomes are more likely to live in open environments than those with small genomes (Kant et al., 2017). In contrast, the strains living in fixed ecological niches (e.g., oral and intestinal tracts) are prone to loss and truncation of some useless gene fragments during evolution, resulting in smaller genome sizes (Duar et al., 2017). Compared with the genome sizes of common *Lactobacillus* strains, ranging from 1.3 to 3.3 Mb, the larger genome of AACE3 suggests that it may live in open environments, with the potential to adapt to a wide ecological niche.

Analysis of the 16S rRNA sequence of AACE3 revealed 99.86–100% identity to *L. chiayiensis* strains, but the similarities to *L. zeae*, *L. paracasei*, *L. casei*, and *L. rhamnosus* strains were all above 99% and > 97% of the interspecific classification threshold (Stackebrandt and Goebel, 1994). In 2008, the International Committee for Systematics of Prokaryotes (ICSP) declared that *L. casei*, *L. paracasei* (subsp. *paracasei* and subsp. *tolerans*) and *L. rhamnosus* are three closely related species in the *L. casei* group for which neither phenotypic nor 16S rRNA gene sequence-based identification methods provide sufficient resolution for accurate identification (Vásquez et al., 2005; Tindall, 2008; Colombo et al., 2014). The same problem has been noted for *L. chiayiensis* AACE3. Next, according to ANI identification, the identity between AACE3 and other *L. chiayiensis* genomes was 98.70–99.81%, which was greater than the 95% classification threshold (Konstantinidis and Tiedje, 2005). The genomic sequence identity with the *L. zeae* strain was 88.56–88.62%, with the *L. paracasei* strain was 79.05–79.09%, and with the *L. rhamnosus* strain was 80.38–80.41%. In addition, in the core genome-based phylogenetic analysis, four strains of *L. chiayiensis* with similar affinities were clustered into one group. The results confirmed that the 16S rRNA gene identity of all four species was above 97%, so it could not be used as an identification criterion. In contrast, whole-genome sequence-based analysis accurately distinguished the four species, suggesting that genome sequences can better reflect the evolutionary relationships of strains compared with 16S rRNA sequences.

Moreover, comparative genomic analysis revealed that AACE3 contains 112 unique genes that were not found in the other three *L. chiayiensis* strains. After KEGG annotation, genes encoding a casein lytic proteinase P (*clpP*, ko: K01358), an oligopeptidase (*pepO,* ko: K07386), and a ferrous iron transport protein A (*feoA*, ko: K04758) were identified. All these genes are closely related to bacterial survival and environmental adaptation. Casein lytic protease P is a central regulator of protein homeostasis in bacteria; *clpP* and its corresponding protease complex have the ability to remove or degrade improperly synthesized, damaged, denatured, aggregated or unwanted proteins from the bacterial cytosol, resulting in the maintenance of a dynamic balance of intracellular proteins under normal bacterial metabolism and stress stimulation (Sauer and Baker, 2011; Culp and Wright, 2017; Glynn, 2017). The oligopeptidase *pepO* hydrolyzes oligopeptides to free amino acids. While most LAB are amino acid auxotrophs, in environments lacking free amino acids (e.g., the milk fermentation process chain), LAB must activate certain gene pathways for hydrolyzing casein in milk to produce free amino acids for growth (Chavagnat et al., 2000). Moreover, the free amino acids produced enter the fermented product and can promote the synthesis of flavor compounds (Gomez et al., 1998). *FeoA* encodes a small basic cytoplasmic protein with 75–85 residues (Cartron et al., 2006), which enhances iron uptake in the presence of *feoB* (Kammler et al., 1993). *BlpA* (ko: K20344) and *comA* (ko: K12292) are related to bacteriocin production and biofilm formation. The *blp* locus is the key to bacteriocin production and immunity in *Streptococcus pneumoniae*, which was activated by *blpA* when protecting the bacteria from foreign bacteriocin invasion (Son et al., 2011). *ComA* was reported to function as a population-sensing regulator, enhancing the formation of bacterial biofilms and the production of bacteriostatic substances (Wang et al., 2016). In summary, *L. chiayiensis* AACE3 has evolved several genes in diverse environments compared with other *L. chiayiensis* strains. These genes may enhance the ability of AACE3 to compete with other microorganisms by producing bacteriocins and forming biofilms in a complex microbial environment. Moreover, the presence of genes such as *clpP*, *pepO*, and *feoA* indicated that AACE3 may have the ability to withstand harsh environmental conditions. These characteristics are directly related to the ecological niche in which AACE3 is found.

Regarding the functional properties during *in vitro* studies. *L. chiayiensis* AACE3 has broad-spectrum inhibitory activity against common gram-positive and gram-negative pathogens. Pathogen antagonism has been considered critical for balancing the normal intestinal microbiota and preventing microecological dysbiosis (Yang et al., 2015). Moreover, it is crucial for LAB application in food supplementation, farming, and other fields. The inhibition of pathogens occurs mainly due to the ability of LAB to produce certain inhibitory substances, such as bacteriocins, H_2_O_2_, lactic acid, and acetic acid (Tambekar et al., 2009). In this study, gene clusters related to bacteriocin production were also detected in the genome of the AACE3 strain. These gene clusters may have the ability to produce blp family class II bacteriocin, sakacin-P, enterolysin_A and enterocin_X. Blp family class II bacteriocin has broad-spectrum activity against *Enterococcus faecalis* and *Streptococcus pyogenes* (Renye et al., 2019). Sakacin-P inhibits the growth of a variety of gram-positive pathogens, especially *Listeria monocytogenes*, a common and persistent pathogen in the food industry (Katla et al., 2001). Enterolysin_A is a heat-resistant bacteriocin with broad-spectrum activity that degrades the cell wall of sensitive cells (Nilsen et al., 2003). Enterocin_X is a novel dual-peptide heat-resistant bacteriocin composed of two antimicrobial peptides (Xα and Xβ), the complementarity of which enhances its antibacterial activity (Hu et al., 2010).

Furthermore, notably, the resistance of bacteria to acid and protease environments and the ability of adhesion are considered critical in various applications, such as probiotic purposes, fermentation industry or food preservation (Monteagudo Mera et al., 2019). During adhesion, bacteria first form a biofilm barrier by self-aggregation (Saito et al., 2019). Cell surface hydrophobicity acts as a driving factor to promote adherence (Haddaji et al., 2015). In this study, *L. chiayiensis* AACE3 had a good survival rate in a simulated artificial gastrointestinal fluid environment and in the presence of 0.3% bile salts. And AACE3 showed good autoaggregation and biofilm formation ability and high cell hydrophobicity. In addition, the antioxidant capacity of LAB has been shown to reduce oxidation-mediated cellular damage and play an important role in the prevention of diabetes, heart disease and intestinal inflammation (Kaushik et al., 2009; Wang et al., 2017). *L. chiayiensis* AACE3 showed good performance in the DPPH radical scavenging test, superoxide anion scavenging test and hydroxyl radical scavenging test. In particular, the antioxidant capacity of CFS was the highest, which indicated that the antioxidant capacity of AACE3 could be attributed to multiple metabolites. The antioxidant capacity of peptides produced by LAB fermentation has been previously reported (Embiriekah et al., 2018). LAB produce a variety of extracellular polysaccharides demonstrated to be associated with free radical scavenging activity (Liu et al., 2011). In summary, the presence of bacteriocin gene clusters, comparative genomic analysis and *in vitro* demonstration of the properties indicate *L. chiayiensis* AACE3 may have strong environmental adaptability. It may have promising applications in antimicrobial therapy and production of antioxidant active substances in adverse environmental production.

## 5. Conclusion

In this study, the whole genome of the LAB strain AACE3 isolated from blueberry fermentation broth was sequenced and identified as *L. chiayiensis*. Genomic characterization and comparative genomic analysis suggested that AACE3 may have the potential to adapt to a wide range of ecological niches. *In vitro* evaluation of the properties indicated that AACE3 has broad-spectrum antibacterial activity and the ability to survive in unfavorable environments, such as in the presence of acid, bile salts, and proteases. In addition, antioxidant tests showed that *L. chiayiensis* AACE3 has good antioxidant capacity and the potential to reduce cellular damage caused by excessive oxidative stress in the host. Meanwhile, the presence of various genes in the genome provided genotypic validation of the results. Overall, this study provides a molecular basis for further investigation of the functional mechanism underlying the properties of this strain and a direction for subsequent application.

## Data availability statement

The datasets presented in this study can be found in online repositories. The names of the repository/repositories and accession number(s) can be found below: https://www.ncbi.nlm.nih.gov/genbank/, CP107523.1.

## Author contributions

X-DL conducted experiments, data sorting, and writing–preparation of original manuscript. Y-CL, R-SY, and XK conducted data analysis, reviewed, and edited the manuscript. W-GX assists in the analysis. FW, Q-LZ, and W-PZ reviewed and revised the manuscript. L-BL designed the study, provided reagents, materials, and revised manuscript. All authors contributed to the article and approved the submitted version.

## Funding

This study was supported by Yunnan Major Scientific and Technological Projects (Grant No. 202202AG050008).

## Conflict of interest

The authors declare that the research was conducted in the absence of any commercial or financial relationships that could be construed as a potential conflict of interest.

## Publisher’s note

All claims expressed in this article are solely those of the authors and do not necessarily represent those of their affiliated organizations, or those of the publisher, the editors and the reviewers. Any product that may be evaluated in this article, or claim that may be made by its manufacturer, is not guaranteed or endorsed by the publisher.

## Footnotes

^1^ https://www.ncbi.nlm.nih.gov/
