# Supplementary material for: Genomic and in-vitro characteristics of a novel strain Lacticaseibacillus chiayiensis AACE3 isolated from fermented blueberry
Source: Front Microbiol. 2023 May 19;14:1168378. doi: 10.3389/fmicb.2023.1168378 (PMC10235500; doi:10.3389/fmicb.2023.1168378)
Supplement: Supplementary file 1 [file Table_1.PDF]

Supplementary Table S1. Genomic features of strains used in this study.

Organism name	Strain	Size (Mb)	GC%	CDS	Accession no.	Level
L. chiayiensis	NCYUAS	2.87	47.1	2648	MSSM01	Contig
L. chiayiensis	BCRC 18859	2.66	47.3	2144	NOXN01	Contig
L. chiayiensis	FBL7	2.86	47.2	2744	CP074378.1	Complete
L. chiayiensis	AACE3	2.87	47.1	2758	CP107523.1	Complete
L.zeae	CECT 9104	3.07	48.0	2753	LS991421.1	Complete
L.zeae	FBL8	3.13	47.8	2967	CP074379.1	Complete
L. paracasei	Zhang	2.90	46.4	2631	CP001084.2	Complete
L. paracasei	CACC 566	3.24	46.2	3015	CP048003.1	Complete
L. rhamnosus	1.0320	2.94	46.7	2622	CP040780.1	Complete
L. rhamnosus	NCTC13764	2.99	46.8	2654	LR134331.1	Complete
