# Supplementary material for: Genomic and in-vitro characteristics of a novel strain Lacticaseibacillus chiayiensis AACE3 isolated from fermented blueberry
Source: Front Microbiol. 2023 May 19;14:1168378. doi: 10.3389/fmicb.2023.1168378 (PMC10235500; doi:10.3389/fmicb.2023.1168378)
Supplement: Supplementary file 2 [file Table_2.PDF]

Supplementary Table S2. The 16S rRNA sequence similarity between strains used in this study.

Strain	1	2	3	4	5	6	7	8	9	10
1	100									
2	100	100								
3	99.86	99.86	100							
4	99.87	99.87	99.93	100						
5	99.68	99.68	99.66	99.68	100					
6	99.61	99.61	99.59	99.62	99.87	100				
7	99.23	99.23	99.19	99.24	99.04	98.98	100			
8	99.23	99.23	99.19	99.24	99.17	99.11	99.87	100		
9	99.04	99.04	98.98	99.05	99.11	99.04	98.85	98.92	100	
10	98.97	98.97	98.92	98.98	99.04	98.98	98.79	98.85	99.94	100

Taxa: 1, *L. chiayiensis* AACE3; 2, *L. chiayiensis* FBL7;3, *L. chiayiensis* BCRC 18859; 4, *L. chiayiensis* NCYUAS; 5, *L. zeae* FBL8; 6, *L. zeae* CECT 9104; 7, *L. paracasei* Zhang; 8, *L. paracasei* CACC 566; 9, *L. rhamnosus* NCTC13764; 10, *L. rhamnosus* 1.0320
