# Supplementary material for: Genomic and in-vitro characteristics of a novel strain Lacticaseibacillus chiayiensis AACE3 isolated from fermented blueberry
Source: Front Microbiol. 2023 May 19;14:1168378. doi: 10.3389/fmicb.2023.1168378 (PMC10235500; doi:10.3389/fmicb.2023.1168378)
Supplement: Supplementary file 4 [file Table_4.PDF]

Supplementary Table S4. Gene clusters encoding the secondary metabolic enzymes in the genome of *L. chiayiensis* AACE3

Region	Gene Position	From	To	Size（nt）	Strand	Annotation
Region 1	locus_00126	133,921	134,529	609	-	HTH-type transcriptional regulator Xre
	locus_00127	134,726	135,010	285	-	hypothetical protein
	locus_00128	135,300	136,169	870	-	Lactococcin-G-processing and transport ATP-binding protein LagD
	locus_00129	136,209	136,895	687	-	hypothetical protein
	locus_00130	137,050	137,274	225	-	hypothetical protein
	locus_00131	137,318	137,521	204	-	hypothetical protein
	locus_00132	137,707	138,003	297	-	hypothetical protein
	locus_00133	138,103	138,312	210	-	Bacteriocin sakacin-P
	locus_00134	139,844	140,824	981	-	hypothetical protein
	locus_00135	140,934	142,001	1,068	-	Microcin C7 self-immunity protein MccF
	locus_00136	142,161	142,412	252	+	hypothetical protein
Region 2	locus_01804	1,804,252	1,804,845	594	+	hypothetical protein
	locus_01805	1,804,982	1,805,269	288	+	Lactococcin-G-processing and transport ATP-binding protein LagD
	locus_01806	1,805,325	1,806,719	1,395	-	hypothetical protein
	**locus_01807**	**1,806,894**	**1,807,004**	**111**	**-**	**hypothetical protein**
	locus_01808	1,808,675	1,808,857	183	+	Bacteriocin sakacin-P
	locus_01809	1,809,117	1,809,803	687	+	hypothetical protein
	**locus_01810**	**1,809,843**	**1,810,430**	**588**	**+**	**ABC-type bacteriocin lantibiotic exporters**
	locus_01811	1,810,651	1,810,914	264	-	hypothetical protein
	locus_01812	1,811,086	1,812,747	1,662	-	putative ABC transporter ATP-binding protein
Region 3	locus_02194	2,208,652	2,209,098	447	+	hypothetical protein
	locus_02195	2,209,095	2,209,658	564	+	hypothetical protein

	locus_02196	2,209,900	2,210,148	249	+	hypothetical protein
	locus_02197	2,210,190	2,211,080	891	+	hypothetical protein
	locus_02198	2,211,257	2,212,234	978	+	Heptaprenyl diphosphate synthase component 2
	locus_02199	2,212,666	2,212,857	192	-	hypothetical protein
	locus_02200	2,212,872	2,213,129	258	-	Blp family class II bacteriocin
	locus_02201	2,214,252	2,215,082	831	+	hypothetical protein
	locus_02202	2,215,359	2,215,856	498	+	hypothetical protein
	locus_02203	2,216,123	2,216,995	873	-	ABC transporter ATP-binding protein NatA
Region 4	locus_02258	2,272,456	2,272,659	204	+	hypothetical protein
	locus_02259	2,273,237	2,273,506	270	+	D-threo-aldose 1-dehydrogenase
	locus_02260	2,273,534	2,274,094	561	+	General stress protein 69
	**locus_02261**	**2,274,048**	**2,274,185**	**138**	**+**	**hypothetical protein**
	locus_02262	2,274,397	2,275,752	1,356	-	Inner membrane protein YjjP
	locus_02263	2,275,867	2,277,246	1,380	-	Lactococcin A secretion protein LcnD
	locus_02264	2,277,259	2,279,451	2,193	-	Lactococcin-G-processing and transport ATP-binding protein LagD
	locus_02265	2,279,760	2,279,906	147	-	hypothetical protein
	locus_02266	2,280,075	2,281,376	1,302	+	hypothetical protein
	locus_02267	2,281,378	2,282,184	807	+	Accessory gene regulator A
	locus_02268	2,282,367	2,282,651	285	-	hypothetical protein
	locus_02269	2,283,378	2,283,569	192	-	hypothetical protein
	locus_02270	2,283,590	2,283,796	207	-	hypothetical protein
	locus_02271	2,283,817	2,284,056	240	-	Blp family class II bacteriocin
	locus_02272	2,284,067	2,284,483	417	-	hypothetical protein
	locus_02273	2,285,071	2,285,229	159	-	hypothetical protein
	locus_02274	2,285,257	2,285,454	198	-	hypothetical protein

	locus_02275	2,285,820	2,286,440	621	-	hypothetical protein
	locus_02276	2,286,519	2,286,914	396	-	hypothetical protein
	locus_02277	2,286,987	2,287,172	186	-	hypothetical protein
	locus_02278	2,287,214	2,287,474	261	-	hypothetical protein
	locus_02279	2,287,822	2,288,013	192	-	hypothetical protein
	**locus_02280**	**2,288,041**	**2,288,217**	**177**	**-**	**hypothetical protein**
Region 5	locus_02331	2,340,321	2,340,638	318	-	hypothetical protein
	locus_02332	2,340,816	2,341,082	267	-	hypothetical protein
	locus_02333	2,341,175	2,341,861	687	-	hypothetical protein
	locus_02334	2,341,858	2,342,031	174	-	hypothetical protein
	locus_02335	2,342,028	2,342,618	591	-	hypothetical protein
	locus_02336	2,342,838	2,343,230	393	-	30S ribosomal protein S9
	locus_02337	2,343,244	2,343,690	447	-	50S ribosomal protein L13
	locus_02339	2,343,877	2,345,244	1,368	-	Lactococcin A secretion protein LcnD
	locus_02340	2,345,250	2,347,427	2,178	-	Lactococcin-G-processing and transport ATP-binding protein LagD
	locus_02341	2,347,558	2,348,157	600	-	HTH-type transcriptional repressor AcnR
	locus_02342	2,348,238	2,349,299	1,062	+	putative ABC transporter permease
	locus_02343	2,349,301	2,350,011	711	+	Lipoprotein-releasing system ATP-binding protein LolD
	locus_02344	2,350,135	2,350,356	222	+	hypothetical protein
	locus_02345	2,350,910	2,351,671	762	+	HTH-type transcriptional activator mta

Bold indicates that this gene is present only in the AACE3 strain and not in other *L. chiayiensis* genomes.
