# Supplementary material for: Genomic and in-vitro characteristics of a novel strain Lacticaseibacillus chiayiensis AACE3 isolated from fermented blueberry
Source: Front Microbiol. 2023 May 19;14:1168378. doi: 10.3389/fmicb.2023.1168378 (PMC10235500; doi:10.3389/fmicb.2023.1168378)
Supplement: Supplementary file 5 [file Table_5.PDF]

Supplementary Table S5. Gene clusters encoding bacteriocins in the *L. chiayiensis* AACE3 genome.

Region	Gene Position	From	To	Size (nt)	Strand	Annotation
Region 1	locus_02260	2,273,534	2,274,094	561	+	General stress protein 69
	locus_02262	2,274,397	2,275,752	1,356	-	Inner membrane protein YjjP
	locus_02263	2,275,867	2,277,246	1,380	-	Lactococcin A secretion protein LcnD
	locus_02264	2,277,259	2,279,451	2,193	-	Lactococcin-G-processing and transport ATP-binding protein LagD
	locus_02266	2,279,760	2,279,906	147	+	hypothetical protein
	locus_02267	2,281,378	2,282,184	807	+	Accessory gene regulator A
	locus_02268	2,282,367	2,282,651	285	-	hypothetical protein
	locus_02269	2,283,378	2,283,569	192	-	hypothetical protein
	locus_02270	2,283,590	2,283,796	207	-	hypothetical protein
	locus_02271	2,283,817	2,284,056	240	-	hypothetical protein
	locus_02272	2,284,067	2,284,483	417	-	hypothetical protein
	locus_02273	2,285,071	2,285,229	159	-	hypothetical protein
	locus_02274	2,285,257	2,285,454	198	-	hypothetical protein
	locus_02275	2,285,820	2,286,440	621	-	hypothetical protein
	locus_02276	2,286,519	2,286,914	396	-	hypothetical protein
	locus_02277	2,286,987	2,287,172	186	-	hypothetical protein
	locus_02278	2,287,214	2,287,474	261	-	hypothetical protein
	locus_02279	2,287,822	2,288,013	192	-	hypothetical protein
	**locus_02280**	**2,288,041**	**2,288,217**	**177**	**-**	**hypothetical protein**
	locus_02281	2,289,090	2,289,845	756	+	Accessory gene regulator A
	locus_02282	2,290,568	2,291,398	831	-	hypothetical protein

	locus_02283	2,291,690	2,292,133	444	-	Putative HTH-type transcriptional regulator YwnA
	locus_02284	2,292,189	2,293,628	1,440	-	Multidrug resistance protein Stp
	locus_02285	2,293,807	2,294,145	339	-	Putative carnobacteriocin-B2 immunity protein
	locus_02286	2,294,446	2,294,622	177	+	hypothetical protein
	locus_02287	2,294,815	2,295,768	954	-	Oxidoreductase YdhF
	locus_02288	2,295,983	2,296,714	732	+	putative oxidoreductase
	locus_02289	2,296,749	2,297,894	1,146	+	putative oxidoreductase
	locus_02290	2,297,899	2,298,216	318	-	hypothetical protein
	locus_02291	2,298,253	2,299,629	1,377	-	Fumarate hydratase class II
	locus_02292	2,299,776	2,300,558	783	-	hypothetical protein
	locus_02293	2,300,878	2,302,485	1,608	-	Divalent metal cation transporter MntH
Region 2	locus_00121	127,940	128,869	930	+	putative ABC transporter ATP-binding protein YxlF
	locus_00122	128,866	129,627	762	+	putative transmembrane protein YxlG
	locus_00123	129,727	130,452	726	+	hypothetical protein
	locus_00124	130,488	131,327	840	+	hypothetical protein
	locus_00125	131,632	133,815	2,184	+	Anaerobic ribonucleoside-triphosphate reductase
	locus_00126	133,921	134,529	609	-	HTH-type transcriptional regulator Xre
	locus_00127	134,726	135,010	285	-	hypothetical protein
	locus_00128	135,300	136,169	870	-	Lactococcin-G-processing and transport ATP-binding protein LagD
	locus_00129	136,209	136,895	687	-	hypothetical protein
	locus_00131	137,318	137,521	204	-	hypothetical protein
	locus_00133	138,103	138,312	210	-	Bacteriocin sakacin-P
	locus_00134	139,844	140,824	981	-	hypothetical protein
	locus_00135	140,934	142,001	1,068	-	Microcin C7 self-immunity protein MccF
	locus_00136	142,161	142,412	252	+	hypothetical protein

	locus_00137	142,660	143,745	1,086	-	D-alanine--D-alanine ligase
	locus_00138	143,939	144,430	492	+	Methylated-DNA--protein-cysteine methyltransferase, inducible
	locus_00139	144,542	145,543	1,002	+	D-2-hydroxyisocaproate dehydrogenase
	locus_00141	145,915	146,265	351	-	hypothetical protein
	locus_00142	146,462	147,919	1,458	+	Adenine permease AdeP
Region 3	locus_01904	1,892,150	1,893,958	1,809	-	ABC transporter permease protein YxdM
	locus_01905	1,893,970	1,894,731	762	-	Bacitracin export ATP-binding protein BceA
	locus_01906	1,895,032	1,896,708	1,677	+	Inner membrane transporter YcaM
	locus_01907	1,896,998	1,897,879	882	+	Diacylglycerol kinase
	locus_01908	1,898,073	1,899,368	1,296	+	GTPase HflX
	locus_01909	1,899,489	1,900,472	984	-	Peptidoglycan-N-acetylglucosamine deacetylase
	locus_01910	1,900,609	1,900,791	183	-	hypothetical protein
	locus_01911	1,901,067	1,902,290	1,224	+	hypothetical protein
	locus_01914	1,903,804	1,905,195	1,392	-	hypothetical protein
	locus_01915	1,905,195	1,905,704	510	-	hypothetical protein
	locus_01916	1,906,060	1,906,902	843	-	dTDP-4-dehydrorhamnose reductase
	locus_01917	1,906,971	1,907,996	1,026	-	dTDP-glucose 4,6-dehydratase
	locus_01918	1,907,999	1,908,571	573	-	dTDP-4-dehydrorhamnose 3,5-epimerase
	locus_01919	1,908,583	1,909,455	873	-	Glucose-1-phosphate thymidylyltransferase 1
	locus_01920	1,909,645	1,911,045	1,401	-	UDP-N-acetylgalactosamine-undecaprenyl-phosphate N-acetylgalactosaminephosphotransferase
	locus_01921	1,911,311	1,912,246	936	-	UDP-N-acetylglucosamine 4-epimerase

Bold indicates that this gene is present only in the AACE3 strain and not in other *L. chiayiensis* genomes.
