# Supplementary figures and images for: Genomic and in-vitro characteristics of a novel strain Lacticaseibacillus chiayiensis AACE3 isolated from fermented blueberry
Source: Front Microbiol. 2023 May 19;14:1168378. doi: 10.3389/fmicb.2023.1168378 (PMC10235500; doi:10.3389/fmicb.2023.1168378)

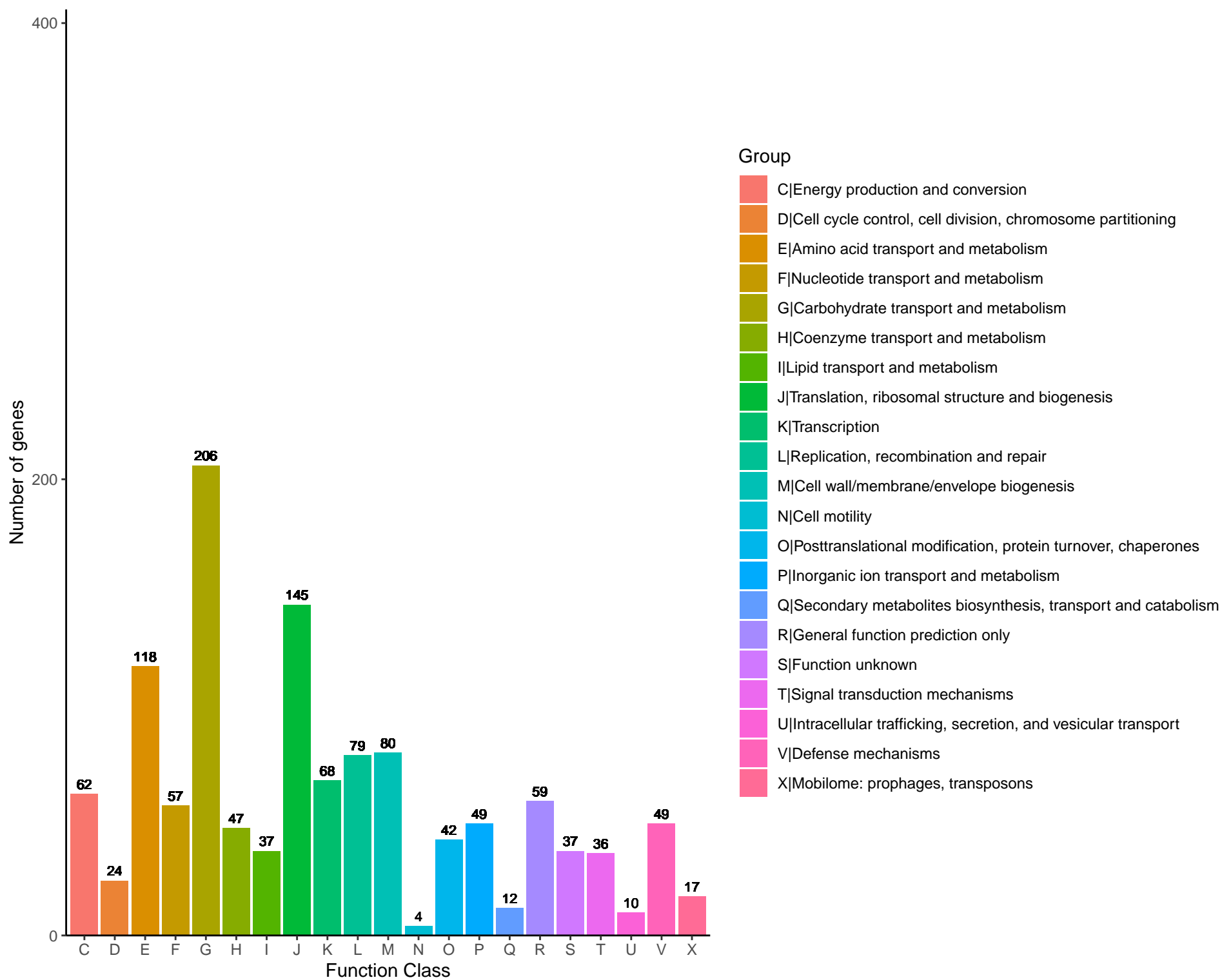

Supplement: Supplementary file 6 [file Image_1.PDF]
